# Yeast Extract Promotes Cell Growth and Induces Production of Polyvinyl Alcohol-Degrading Enzymes

**DOI:** 10.4061/2011/179819

**Published:** 2011-10-01

**Authors:** Min Li, Xianyan Liao, Dongxu Zhang, Guocheng Du, Jian Chen

**Affiliations:** ^1^The Key Laboratory of Industrial Biotechnology, Ministry of Education, School of Biotechnology, Jiangnan University, 1800 Lihu Road, Wuxi 214122, China; ^2^College of Life Science and Technology, Inner Mongolia Normal University, Huhhot 010022, China

## Abstract

Polyvinyl alcohol-degrading enzymes (PVAases) have a great potential in bio-desizing processes for its low environmental impact and low energy consumption. In this study, the effect of yeast extract on PVAases production was investigated. A strategy of four-point yeast extract addition was developed and applied to maximize cell growth and PVAases production. As a result, the maximum dry cell weight achieved was 1.48 g/L and the corresponding PVAases activity was 2.99 U/mL, which are 46.5% and 176.8% higher than the control, respectively. Applying this strategy in a 7 L fermentor increased PVAases activity to 3.41 U/mL. Three amino acids (glycine, serine, and tyrosine) in yeast extract play a central role in the production of PVAases. These results suggest that the new strategy of four-point yeast extract addition could benefit PVAases production.

## 1. Introduction

Polyvinyl alcohol (PVA), a water-soluble synthetic polymer, has commercial applications in the adhesive, paper-coating, and textile industries [1–3]. The material is also of interest for packaging applications, such as bags for dyeing shops, packaging of household items, packaging of monodoses for powders and solid tablets, as well as for liquid concentrates, monodose packaging of chemicals for agriculture such as pesticides, and herbicides [4]. However, it is one of the major water pollutants in industrial wastewater, especially in textile factory wastewater. PVA is not easily decomposed in the environment. Therefore, biological methods were investigated as a means of degrading PVA [5–8]. 

Researchers worldwide have focused their attention on new microbial isolates [9, 10], purification of PVA-degrading enzymes (PVAases) [11–13], and PVA biodegradation mechanisms [13]. PVA has been found to be the only vinyl polymer that can be utilized by some bacteria as a carbon and energy source [11, 14]. There have been studies on the mechanisms of PVA biodegradation. Sakai et al. reported that PVA was degraded by successive reactions of secondary alcohol oxidase and *β*-diketone hydrolase (oxidized PVA hydrolase) from *Pseudomonas *sp. [15]; this suggests the possibility of formation of *β*-diketone structures during the reaction of secondary alcohol oxidase on PVA. *β*-Diketone hydrolase catalyzes the hydrolysis of *β*-diketone to form a methyl ketone and a carboxylic acid. A different pathway for PVA degradation in *Pseudomonas *sp. VM15C is the combination of a PQQ-dependent PVA dehydrogenase (PVADH) and oxidized PVA hydrolase (OPH) (an enzyme that acts on an oxidized PVA) [16]. PVADH and OPH constitute an enzyme system for the cleavage of PVA molecules. PVADH introduces *β*-diketone groups into the PVA molecule, and OPH subsequently hydrolyzes these *β*-diketone groups in oxidized PVA.

Actually, the physiological characteristic of PVAases production by microorganisms in a submerged culture remains sketchy. Our previous studies found that yeast extract is a rate-limiting factor for PVAases production [17]. Other work also relied on yeast extract as an energy source in culturing PVA-degrading bacteria, and the critical component has yet to be identified [11, 18]. 

Therefore, the intention of this work is to quantify the effect of yeast extract on the PVAases fermentation. This study uses an effective mixed culture screened by Chen et al. which is capable of high PVA-degrading enzyme production [5]. The observation that yeast extract stimulates cell growth and PVAases production prompted us to conduct further research on the components in yeast extract that are responsible for PVAase production. To our knowledge, this is the first study that shows yeast extract to play a clear promoting role in the production of PVAases. 

## 2. Methods

### 2.1. PVA Materials

PVA 1799 with a 1700 polymerization degree and 99.0% saponification degree was bought from Sichuan Vinyl Factory (Mianyang, China).

### 2.2. Microorganisms and Culture Media

The strains used in this study were isolated from PVA-rich soil sampled at Pacific Textile Co. (Jiangsu Province, China), and the ability of the mixed culture in utilizing PVA was investigated in our previous study [9]. The composition of seed and fermentation media was as follows (g/L): PVA1799 5, yeast extract 2, K_2_HPO_4_·3H_2_O 2, KH_2_PO_4_ 0.25, MgSO_4_·7H_2_O 0.1, CaCl_2_ 0.025, FeSO_4_·7H_2_O 0.05, NaCl 0.02.

### 2.3. Shake Flask Culture Conditions

Flask culture experiments were performed in 500 mL flasks each containing 50 mL medium after inoculating with 10% (v/v) of seed culture. The mixed culture was grown in flasks at 30°C and 200 rpm for 36 h.

### 2.4. Batch Fermentation in 7 L Stirred Fermentor

Fermentations were carried out in a fermentor (KF-7 L, Korea Fermentor Co., Inchon, Korea) with 4 L fermentation medium and an inoculum size of 10% (v/v). The pH was controlled automatically at 7.2 by adding 2 M NaOH or 2 M HCl solution. Aeration rate was 5 L/min and agitation speed was controlled at 300 rpm.

### 2.5. Yeast Extract Addition

The type of “yeast” used for the extracts is *Saccharomyces cerevisiae*. The reagents were purchased from Sangon Biotech (Shanghai) Co., Ltd.

Continuous yeast extract addition: yeast extract was fed to the fermentor continuously at a rate of 0.02, 0.04, or 0.06 g/L/h. Feeding of yeast extract started at 18 h of fermentation and stopped at 30 h.

Single-yeast extract addition: yeast extracts (1, 2, or 3 g/L) were added to the culture broth in flasks at various cultivation times (0, 6, 12, 18, and 24 h).

Repeated yeast extract addition: 2-, 3-, and 4-point additions of yeast extract with a final cumulative concentration of 2 g/L were carried out at different times (18, 20, 22, and 24 h) in flasks.

### 2.6. Amino Acids Addition

To find out which amino acids in yeast extract are the key ingredients for the production of PVAases, a mixture of 17 kinds of amino acids (at concentrations identical to those found in yeast extract) was added into the medium. One of the 17 amino acids was omitted each time. Cells were cultured with NH_4_Cl as the nitrogen source, which leads to no detectable PVAases activity.

### 2.7. Analytical Methods

Cells were harvested by centrifugation at 15,000× g and 4°C for 20 min. Dry cell weight (DCW) was determined after drying cells at 105°C to a constant weight.

Quantitative determination of PVA concentration in culture broth was carried out by spectrophotometric analysis after addition of boric acid and iodine solutions according to the procedure described by Finley [19]. 

The assay of PVAases activity was based on the methods described by Mori et al. [20]. One unit of enzyme activity is defined as a decrease in absorbance at 1 × 10^−3^ per min at 690 nm under the specified conditions and is expressed as the total degradation activity for PVA.

To understand the changes of amino acids in the cultivation media during the bacterial growth, the analysis of the amino acids composition in the media was done according to the methods described by Jae-Young et al. [21]. Samples were analyzed with an amino acid analyzer (Agilent1100, USA). All analyses were done in duplicates, and the average value was reported.

## 3. Results

### 3.1. Batch Fermentation in Fermentor

**Figure 1:**
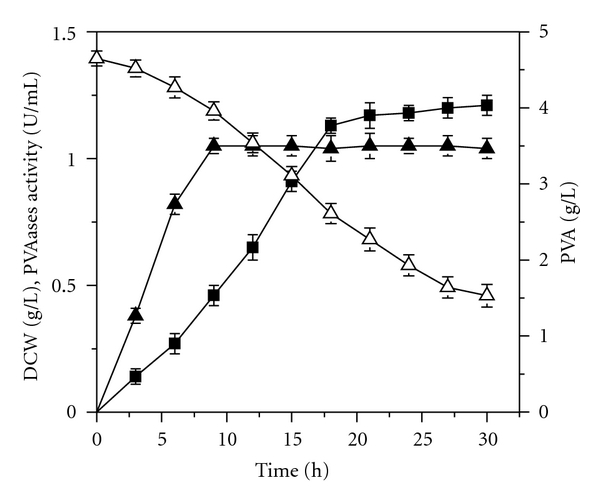
Cell growth and PVAases production in fermentor. PVA (△); DCW (▲); PVAases activity (■).

Yeast extract contains abundant of vitamins, minerals, and amino acids, which are necessary for cell growth and PVAases synthesis [22]. Batch fermentation with yeast extract as nitrogen source in 7 L fermentor for PVAases production is shown in Figure 1. Cell growth stopped after 9 h of cultivation, and a maximum DCW of 1.08 g/L was obtained. After that, PVAases activity increased continually to 1.23 U/mL at 30 h. PVA was continuously degraded to 1.53 g/L at the end of fermentation. In our previous studies, it was found that the addition of yeast extract was not only advantageous to the growth of the mixed culture, but also propitious to PVAases synthesis [17]. With yeast extract as nitrogen source, we further investigate effects of different addition strategy of yeast extract on cell growth and the production of PVAases by feeding at a constant rate, single bolus addition, and repeated additions.

### 3.2. Effect of Continuous Yeast Extract Feeding at Constant Rates on Cell Growth and PVAases Production in Fermentor

PVAases are increasing rapidly until 18 h, and then keeping a very slow increment. Feeding of yeast extract was therefore started at 18 h of fermentation and stopped at 30 h. 

PVAase activity reflects the total enzyme production capacity of all cells, while specific PVAase activity reflects enzyme production capacity of unit cell. The increase of the enzyme production capacity can be obtained by improving the specific PVAase activity [23]. The kinetic parameter of the specific PVAase activity was obtained from the following equation [24]:

(1) $$q_{P}=\frac{1}{X}\;\frac{dP}{dt}=\frac{1}{X}\;\underset{\Delta t\to 0}{\lim\;}\frac{\Delta P}{\Delta t}.$$

**Figure 2:**
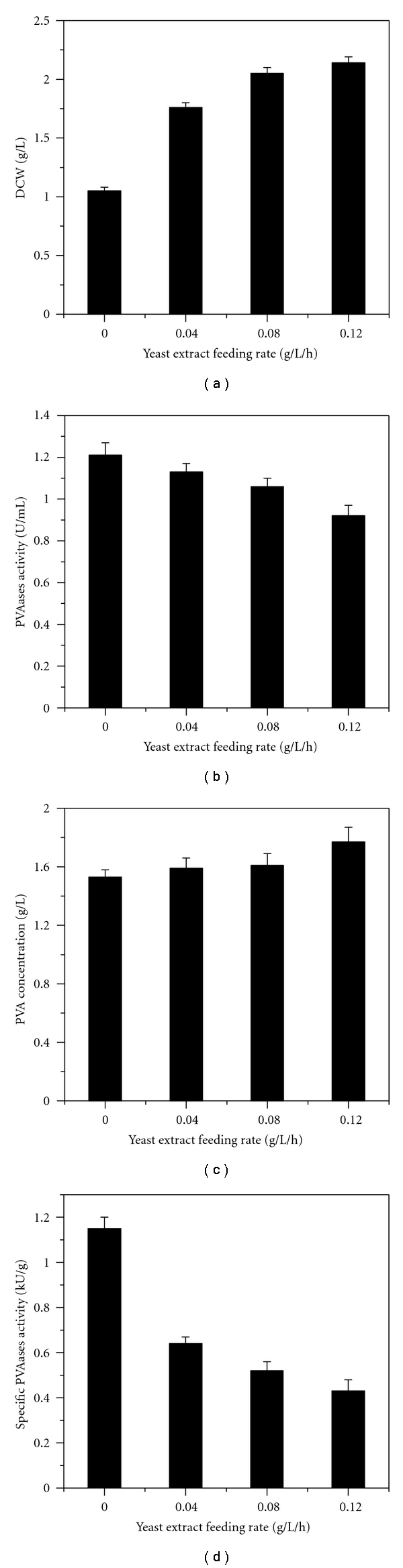
Effects of continuous yeast extract feeding on cell growth and PVAases production in fermentor.

Figure 2 shows the effect of yeast extract feeding rate (0.04, 0.08, and 0.12 g/L/h) on cell growth and PVAases production. Cell growth was obviously enhanced with increasing yeast extract feeding rate as compared to the control. However, the highest PVAases activity achieved in each case was lower than the control. Meanwhile, all specific PVAases activities were lower than the control and were negatively affected by feeding rates of yeast extract. As a result, a higher DCW and lower PVAases activity were observed.

Based on the above results, we conclude that it is unsuitable to adopt a continuous yeast extract feeding method to improve PVAases production. It is reasonably believed that addition times and concentrations of yeast extract could affect cell growth and PVAases synthesis. So it is necessary to investigate the timing and strength of yeast extract addition. The following studies examine the effect of repeated yeast extract addition on cell growth and PVAases production.

### 3.3. Effects of Single Bolus Yeast Extract Addition on Cell Growth and PVAases Production in Shaker Flasks

**Figure 3:**
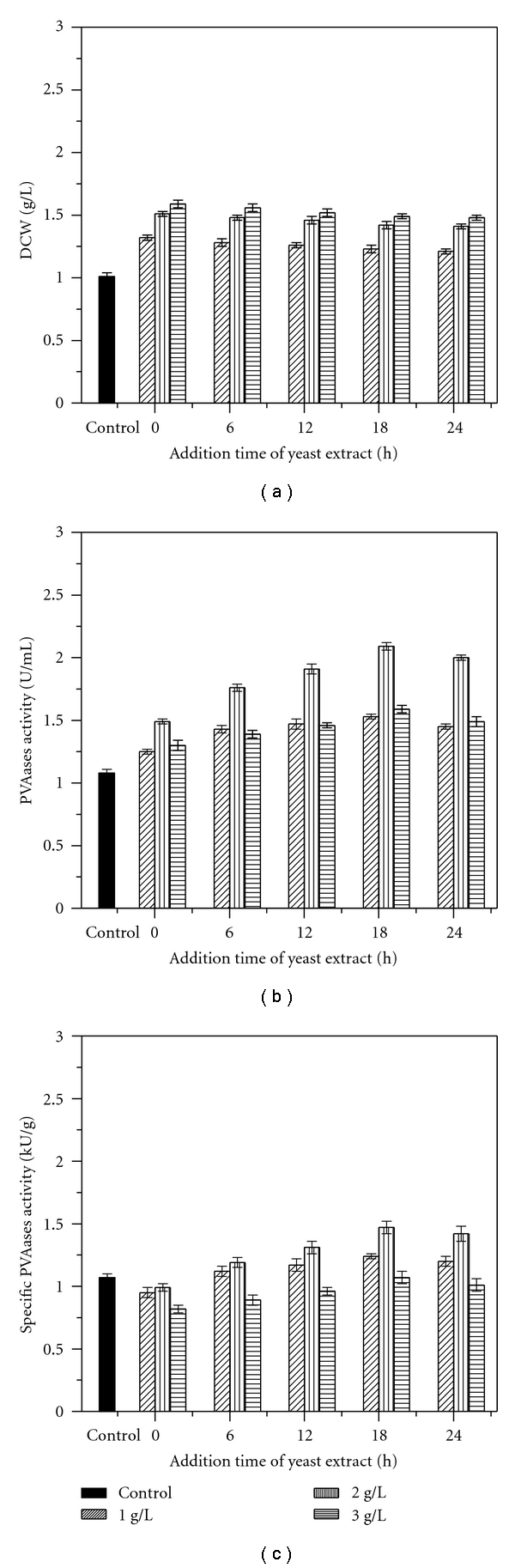
Effect of single yeast extract addition on cell growth and PVAases production with yeast extract concentrations of 1 g/L, 2 g/L, and 3 g/L.

To obtain higher PVAases activity, yeast extract with concentrations of 1, 2, and 3 g per liter of fermentation broth was added separately to the culture media, at 0, 6, 12, 18, and 24 h of cultivation. DCW and PVAases activity are shown in Figure 3. Single yeast extract addition can effectively promote cell growth and PVAases accumulation simultaneously. And the promotion degree accelerated with delaying of yeast extract addition time. 

Irrespective of concentration, yeast extract addition at 18 h of fermentation resulted in highest PVAases production. Since PVAases activity ceased to increase after 18 h of fermentation in control, the absence of yeast extract in culture media limited PVAases synthesis. Addition at this time satisfied the necessary of cell growth and PVAases production. For instance, with 2 g/L yeast extract addition at 18 h of cultivation, maximum PVAases activity was 2.09 U/mL, and the corresponding specific PVAases activity was 1.47 kU/g. 

Moreover, the highest PVAases activity can be achieved with yeast extract concentration of 2 g/L among all three different concentrations. In addition, the maximum PVAases activity achieved with addition of 1 g/L yeast extract was higher than that of 3 g/L. Because neither too little nor too much yeast extract at one time is optimal, the following studies investigate whether repeated yeast extract additions spaced over time can bring about higher PVAases production than single bolus addition. Our previous results showed that PVAases activity decreased with yeast extract addition after 24 h; yeast extract was therefore added between 18 and 24 h for PVAases production. 

### 3.4. Effects of Repeated Additions of Yeast Extract on PVAases Production in Shaker Flasks

Yeast extract was added over two time points, three points and four points with a total level of 2 g/L between 18 and 24 h, and the results are shown in Table 1. DCW and corresponding PVAases activity increased with the number of yeast extract additions. With four-point yeast extract addition (0.5 g/L of yeast extract each time added at 18 h, 20 h, 22 h, and 24 h), the highest PVAases activity at 2.99 U/mL and the corresponding specific PVAases activity at 2.02 kU/g were achieved. These were 43.1% and 37.4% higher than single addition. Because PVAases activity of 2.88 U/mL obtained in three-point yeast extract addition was only slightly lower than that in four-point addition, more frequent additions will unlikely lead to higher PVAases activity. PVAases activity (2.99 U/mL) obtained by four-point yeast extract addition was 176.8% higher than the control. Therefore, this new strategy can enhance batch PVAases production.

### 3.5. Applications of Multiple Points of Yeast Extract Addition Strategies to Fermentor

To prove effectiveness of optimized four-point yeast extract addition in flasks, we further applied this strategy to batch fermentation in a 7 L fermentor.  Table 2 compares PVAases activities between single addition, two-point addition, three-point addition, and four-point addition of yeast extract. In control, DCW reached a maximum of 1.08 g/L after 9 h, and PVAases activity reached 1.23 U/mL after 30 h. In contrast, with four-point yeast extract addition, PVAases activity reached a maximum of 3.41 U/mL at the end of fermentation at 30 h.

In four-point yeast extract addition, PVAases activity obtained in flasks (2.99 U/mL) was lower than that achieved in a fermentor (3.41 U/mL). Because of a lack of pH control in shaker flasks, a lower PVAases activity in flasks suggests that besides yeast extract addition, pH plays a role in PVAase production.

### 3.6. Role of Amino Acid for the Production of PVAases

Enhanced PVAases activity with the addition of yeast extract suggests that yeast extract is involved in the synthesis of PVAases. However, the specific component(s) in yeast extract responsible for enhanced PVAases synthesis remains unknown. To identify this, we focused on amino acids, which are the major components of yeast extract. We examined the effect of each of the 17 amino acids that are present in yeast extract (data not shown) on cell growth and PVAases production.

As shown in Table 3, PVAases activity decreased by 78.7%, 66.9%, and 83.1% in the absence of glycine, tyrosine, and serine, respectively; whereas, the absence of other amino acids had relatively little or no effect. It is clear that glycine, tyrosine, and serine are the key amino acids in PVAases synthesis. Table 3 also shows that DCW and PVAases activity with the addition of yeast extract were 10.4% and 10.5%, respectively, higher than those with the addition of the amino acid mixture. This discrepancy suggests that components in yeast extract other than amino acids may play a role in improving DCW and PVAases activity.

**Figure 4:**
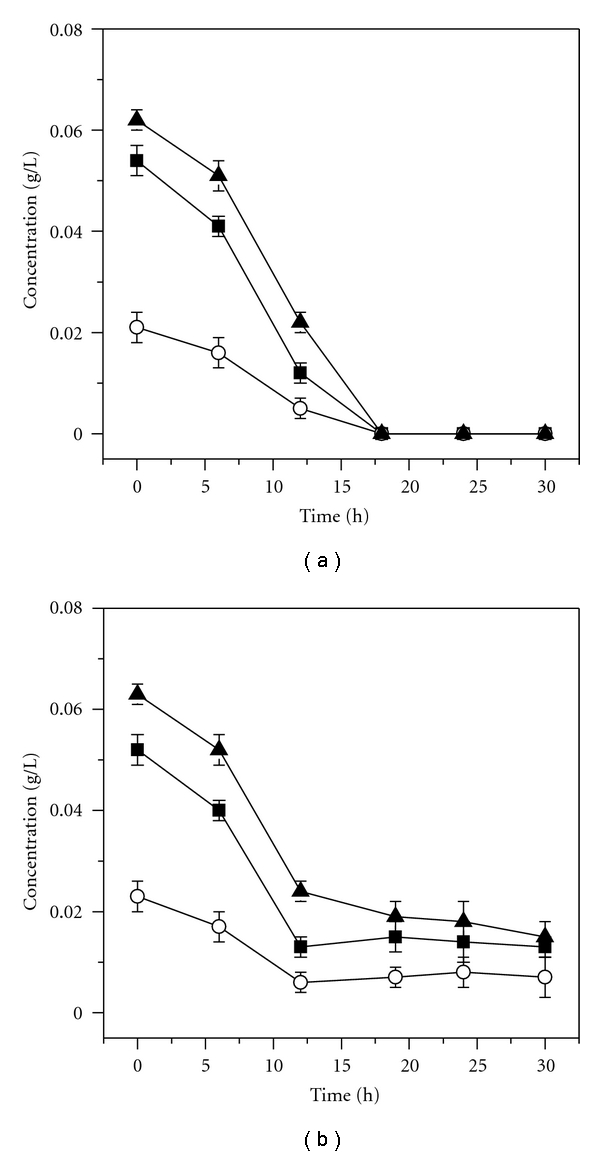
Profiles of glycine (■), tyrosine (○), and serine (▲) during PVAases production with (b) and without (a) four-point yeast extract addition.

We profiled glycine, tyrosine, and serine during the PVAases production process with and without four-point yeast extract addition.  Figure 4(a) shows that these three amino acids were completely exhausted at 18 h without four-point yeast extract addition. In contrast to the control, these three amino acids keep a stable level from 18 h to the end of fermentation with four-point yeast extract addition (Figure 4(b)). This further confirms that depletion of glycine, tyrosine, and serine limited PVAases synthesis.

## 4. Discussion

Yeast extract is usually used as growth stimulants or growth factors for bacteria [5, 23]. Effects of yeast extract on cell growth and PVAases production by a mixed culture were first investigated in this study. 

PVAases are enzymes inducible by PVA [25], and they are synthesized mostly during the stationary phase of cell growth (Figure 1). During cell growth phase, the nutrients are consumed mainly for cell growth but not for enzyme synthesis. Therefore, yeast extract impacted PVAases synthesis heavily when added at the beginning of fermentation, as opposed to the stationary phase (Figure 3). However, a shot of yeast extract added at a high concentration (3 g/L) does not impact as positively on PVAases production as a shot added at a lower concentration (2 g/L) (Figure 3). Excessive yeast extract may inhibit PVAases synthesis. As expected, the strategy of adding yeast extract over multiple number of times maximizes PVAases activity, presumably by avoiding generating inhibitory levels of amino acids. Application of this strategy to 7 L fermentor was also feasible (Figure 4). An increased PVAases activity with the addition of yeast extract indicated that the components in yeast extract enhanced the synthesis of PVAases.

Threonine was proven to be essential for the strain *Penicillium *sp. to produce PVAases [26]. In our study, it is found that glycine, tyrosine, and serine significantly stimulate the production of PVAases. PVAases are not one but are a collection of PVA-degrading enzymes, such as PVA oxidase, PVA dehydrogenase, and PVA hydrolase. Study on the purification of PVAases will help us to illustrate how these three amino acids accelerate the production of PVAases in the mixed culture.

Thus, several amino acids, including tyrosine, glycine, and serine, had a promoting role in PVAases production. Although tyrosine has been reported by Fujita for its role in PVAases synthesis [22], glycine and serine have not been previously reported for their roles in the production of PVAases. This result suggests that these amino acids may directly participate in the synthesis of PVAases. 

## Figures and Tables

**Table 1:** Effects of multiple yeast extract addition on cell growth and PVAases production in flask. Number (1) 2 g yeast extract per liter of culture broth added at 18 h with a volume of 6 mL; number (2) 1 g yeast extract per liter of culture broth added at 18 h and 20 h with a volume of 3 mL each time; number (3) 0.67 g yeast extract per liter of culture broth added at 18 h, 20 h, and 22 h with a volume of 2 mL each time; number (4) 0.50 g yeast extract per liter of culture broth added at 18 h, 20 h, 22 h, and 24 h with a volume of 1.5 mL each time.

No.	Addition time				Experimental results		
	18 h	20 h	22 h	24 h	DCW (g/L)	PVAases activity (U/mL)	Specific PVAases activity (kU/g)
	Concentration of yeast extract (g/L)						
0	0 (Control)				1.01 ± 0.03	1.08 ± 0.02	1.07 ± 0.02
1	2				1.42 ± 0.03	2.09 ± 0.06	1.47 ± 0.05
2	1	1			1.44 ± 0.03	2.33 ± 0.07	1.62 ± 0.07
3	0.67	0.67	0.67		1.47 ± 0.05	2.88 ± 0.08	1.96 ± 0.06
4	0.5	0.5	0.5	0.5	1.48 ± 0.04	2.99 ± 0.09	2.02 ± 0.07

**Table 2:** Effects of multiple yeast extract addition on cell growth and PVAases production in fermentor. Number (1) 2 g yeast extract per liter of culture broth added at 18 h with a volume of 96 mL; number (2) 1 g yeast extract per liter of culture broth added at 18 h and 20 h with a volume of 48 mL each time; number (3) 0.67 g yeast extract per liter of culture broth added at 18 h, 20 h, and 22 h with a volume of 32 mL each time; number (4) 0.50 g yeast extract per liter of culture broth added at 18 h, 20 h, 22 h, and 24 h with a volume of 24 mL each time.

No.	Addition time				Experimental results		
	18 h	20 h	22 h	24 h	DCW (g/L)	PVAases activity (U/mL)	Specific PVAases activity (kU/g)
	Concentration of yeast extract (g/L)						
0	0 (Control)				1.08 ± 0.07	1.23 ± 0.10	1.14 ± 0.10
1	2				1.52 ± 0.06	2.31 ± 0.08	1.52 ± 0.12
2	1	1			1.54 ± 0.05	2.66 ± 0.09	1.73 ± 0.07
3	0.67	0.67	0.67		1.56 ± 0.08	3.27 ± 0.11	2.09 ± 0.13
4	0.5	0.5	0.5	0.5	1.58 ± 0.07	3.41 ± 0.13	2.15 ± 0.09

**Table 3:** Role of amino acids on DCW and PVAases.

Nitrogen source	DCW (g/L)	PVAases activity (U/mL)	Specific PVAases activity (kU/g)
Yeast extract	1.38 ± 0.03	1.97 ± 0.05	1.43 ± 0.04
AA mixture^a^	1.25 ± 0.02	1.70 ± 0.04	1.36 ± 0.03
AA mixture (Ala^−^)^b^	1.24 ± 0.04	1.62 ± 0.06	1.31 ± 0.03
AA mixture (Val^−^)^b^	1.23 ± 0.04	1.64 ± 0.05	1.33 ± 0.04
AA mixture (Leu^−^)^b^	1.24 ± 0.02	1.69 ± 0.04	1.36 ± 0.05
AA mixture (Ile^−^)^b^	1.26 ± 0.04	1.66 ± 0.04	1.32 ± 0.03
AA mixture (Phe^−^)^b^	1.25 ± 0.02	1.64 ± 0.05	1.31 ± 0.03
AA mixture (Met^−^)^b^	1.25 ± 0.04	1.72 ± 0.06	1.38 ± 0.04
AA mixture (Pro^−^)^b^	1.24 ± 0.03	1.70 ± 0.04	1.37 ± 0.04
AA mixture (Gly^−^)^b^	1.20 ± 0.04	0.35 ± 0.01	0.29 ± 0.01
AA mixture (Ser^−^)^b^	1.21 ± 0.02	0.28 ± 0.01	0.23 ± 0.01
AA mixture (Thr^−^)^b^	1.21 ± 0.03	1.62 ± 0.04	1.34 ± 0.04
AA mixture (Cys^−^)^b^	1.22 ± 0.04	1.63 ± 0.05	1.34 ± 0.04
AA mixture (Tyr^−^)^b^	1.23 ± 0.02	0.55 ± 0.04	0.45 ± 0.02
AA mixture (His^−^)^b^	1.25 ± 0.03	1.68 ± 0.04	1.34 ± 0.03
AA mixture (Lys^−^)^b^	1.24 ± 0.03	1.64 ± 0.05	1.32 ± 0.04
AA mixture (Arg^−^)^b^	1.26 ± 0.02	1.67 ± 0.04	1.33 ± 0.03
AA mixture (Asp^−^)^b^	1.23 ± 0.04	1.67 ± 0.05	1.36 ± 0.04
AA mixture (Glu^−^)^b^	1.21 ± 0.03	1.61 ± 0.04	1.33 ± 0.04

^a^AA mixture: amino acids mixture contains 17 amino acids, which were detected in yeast extract, respectively, Alanine (Ala), Valine (Val), Leucine (Leu), Isoleucine (Ile), Phenylalanine (Phe), Methionine (Met), Proline (Pro), Glycine (Gly), Serine (Ser), Threonine (Thr), Cysteine (Cys), Tyrosine (Tyr), Histidine (His), Lysine (Lys), Argnine (Arg), Aspartic (Asp), and Glutamic (Glu).

^b^free of which.
